# MYC-dependent MiR-7-5p regulated apoptosis and autophagy in diffuse large B cell lymphoma by targeting AMBRA1

**DOI:** 10.1007/s11010-024-04946-w

**Published:** 2024-02-23

**Authors:** Cuifen Zhang, Ke Wang, Jiahao Tao, Chuangjie Zheng, Linzhu Zhai

**Affiliations:** 1 Guangzhou University of Chinese Medicine, Guangzhou, 510407 China; 2 Lingnan Medical Research Center, Guangzhou University of Chinese Medicine, Guangzhou, 510407 China; 3 Cancer Center, Departments of Radiation Oncology, The First Affiliated Hospital of Guangzhou University of Chinese Medicine, No. 16 Jichang Road, Baiyun District, Guangzhou, 510405 People’s Republic of China

**Keywords:** Diffuse large B-cell lymphoma (DLBCL), miR-7-5p, AMBRA1, Autophagy, Apoptosis

## Abstract

Diffuse large B-cell lymphoma (DLBCL) is the leading cause of mortality from invasive hematological malignancies worldwide. MicroRNA-7-5p (miR-7-5p) has been shown to be a tumor suppressor in several types of tumors. However, its role in DLBCL is not fully understood. This study explored the role of miR-7-5p in the progression of DLBCL and pursued the underlying mechanism. Quantitative real-time PCR and transfection of miRNA mimic and inhibitors were used to assess the effects of miR-7-5p on autophagy and apoptosis in SU-DHL-4 and SU-DHL-10 cells. Dual-luciferase reporter assay was used to identify target genes of miR-7-5p. Immunofluorescence, flow cytometry, and western blotting (WB) were performed to explore the underlying mechanism and downstream pathways of miR-7-5p and AMBRA1 in DLBCL cells. MiR-7-5p was upregulated in DLBCL cells. Luciferase reporter assays implicated AMBRA1 as a downstream target of miR-7-5p in DLBCL. WB and flow cytometry showed that an increase in miR-7-5p level and a decrease in AMBRA1 expression led to a decrease in autophagy and apoptosis-related protein expression. Furthermore, miR-7-5p prevented c-MYC dephosphorylation through AMBRA1 downregulation. On the contrary, c-MYC increased the expression of miR-7-5p, thereby establishing positive feedback on miR-7-5p transcription. The addition of hydroxychloroquine, an autophagy inhibitor, reduced autophagy and increased apoptosis in DLBCL cells. In *vivo* experiments further proved that the increase of miR-7-5p played a regulatory role in the expression of downstream AMBRA1 and c-MYC. These results demonstrate that c-MYC-dependent MiR-7-5p suppressed autophagy and apoptosis by targeting AMBRA1 in DLBCL cells. MiR-7-5p also suppressed autophagy and apoptosis by targeting AMBRA1 in DLBCL cells. Therefore, these data suggest that targeting miR-7-5p may be a promising strategy in DLBCL therapy.

**Supplementary Information:**

The online version contains supplementary material available at 10.1007/s11010-024-04946-w.

## Introduction

Non-Hodgkin’s lymphoma (NHL) is the most common hematological malignancy worldwide [1].

Diffuse large B-cell lymphoma (DLBCL) is the most common invasive non-Hodgkin’s lymphoma [2], accounting for 25–30% of adult non-Hodgkin’s lymphoma in western countries and approximately 30–40% of all NHL [1, 3–5]. NHL is a heterogeneous disease with different prognoses. For example, the prognosis of Germinal-center B-cell-like (GCB) DLBCL is significantly better than that of non-GCB DLBCL [6].

MicroRNA (miRNA) is a highly conserved endogenous noncoding small molecule RNA with a length of approximately 18 to 21 nucleotides. MiRNAs can maintain the proper regulation of cellular processes such as cell proliferation, metabolism, and protein synthesis. Downregulation of miRNA can affect cell growth and biosynthesis, leading to the development and progression of tumors [7, 8]. Many research studies have identified the importance of miRNA expression regulatory mechanisms in leukemia and lymphoma. Cimmino et al. confirmed that miR-15 and miR-16 induced apoptosis through targeting B cell lymphoma 2 (BCL2) in leukemia [9]. MiRNA-340-5p was shown to mediate the function and invasive promotion of tumor infiltrating CD8 + T lymphocytes in human DLBCL [10]. Nodal marginal zone lymphoma showed increased expression of miR-221, miR-223, and let-7f, while follicular lymphomas were found to strongly express miR-494 [11].

MicroRNA-7 (miR-7) is a type of miRNA that plays diverse roles in physiological and pathological conditions. MiR-7-5p is the most studied miRNA sequence in this family [12]. According to relevant studies, miR-7-5p expression is deficient in breast cancer [13], melanoma [14], hepatocellular carcinoma [15], and glioblastoma [16]. Recent studies have shown that miR-7-5p acts to down regulate the transcription and translation of direct or indirect tumor promoters, inhibiting proliferative and invasive malignant phenotypes [17]. However, to our knowledge, the potential mechanism of miR-7-5p in the progression of DLBCL remains unclear.

Autophagy/beclin 1 regulator 1 (AMBRA1) is a newly identified autophagy-related gene (ATG), and its key regulatory role in autophagy has been determined [18]. AMBRA1 was shown to regulate autophagosome formation by interacting with Beclin1 (autophagy-related protein encoded by becn1 gene) through its target lipid kinase type III phosphatidylinositol 3-kinase [19, 20]. In addition, AMBRA1 enhanced autophagy via a positive feedback loop through regulation of ubiquitin-mediated ulk1 stabilization [21, 22]. In addition to playing a central role in autophagy, Beclin 1 interacts with a variety of cofactors (atg14l, UVRAG, Bif-1, Rubicon, and AMBRA1) to form a 1-vps34-vps15 core complex, thus inducing autophagy. In addition, the BH3 domain of Beclin 1 binds to and is inhibited by Bcl-2 or BCL XL. This interaction can be disrupted by phosphorylation of Bcl-2 and Beclin 1 or ubiquitination of Beclin 1. Further, it was found that caspase-mediated cleavage of Beclin 1 promoted crosstalk between apoptosis and autophagy [23]. AMBRA1 is indispensable in the intersection of autophagy and apoptosis [24]. It was found that AMBRA1 can negatively regulate apoptosis and growth in prostate cancer and breast cancer cells and is related to AMBRA1-mediated autophagy promotion [25, 26]. Further, it was reported that AMBRA1 in solid tumors regulated autophagy or apoptosis through mTORC1 (upstream) and c-MYC (downstream) [27, 28]. However, the function of AMBRA1 in DLBCL development is poorly understood.

Given the importance of miR-7-5p and AMBRA1 in tumor initiation and development, the current study investigated whether miR-7-5p regulates AMBRA1, and if miR-7-5p is MYC-dependent in DLBCL. Furthermore, the effects of miR-7-5p and AMBRA1 expression on autophagy and apoptosis were examined. We also explored whether inhibition of AMBRA1-induced autophagy had an effect on apoptosis in DLBCL.

## Materials and methods

### Cell lines

All DLBCL cell lines were purchased from Cellcook. HMy2.CIR cells were cultured in Iscove’s Modified Dulbecco Medium (Gibco cat:12,440) supplemented with 10% fetal bovine serum, 100 mg/mL streptomycin, and 100 units/mL penicillin. SU-DHL-2, 4, 6, 10 cell lines were similarly cultured in RPMI 1640 (CellCook cat:CM2006) supplemented with 10% fetal bovine serum, 100 mg/mL streptomycin, and 100 units/mL penicillin in a CO2-humidified incubator at 37 °C.

### Animals and ethics statement

Sixteen female SCID mice weighing 20–22 g were obtained from Guangdong Sijia Jingda Biotechnology Co., Ltd and randomly divided into two groups (the control group and the miR-7-5p mimic group). All mice were maintained in a specific pathogen-free room with free access to water and standard laboratory chow. The room was supplied with a standard light–dark (12 h light/12 h dark) cycle with the temperature kept at 22–24 °C. All animal experiments were conducted with the approval of the Institutional Animal Care and Use Committee of Experimental Animal Center of Guangzhou University of Traditional Chinese Medicine.

### Quantitative reverse-transcription PCR (qRT-PCR)

RNA was extracted using Trizol and an RNA Extraction Kit (MRC, TR118-500). After extracting total RNA, M-MLVReverseTranscriptase (Promega, M1705) and ChamQSYBRqPCRMasterMix (Vazyme, Q341-03) were used to generate cDNA from RNA following the manufacturer’s protocol. The expression levels of miR-7-5p and AMBRA1 were normalized to the expression of U6 and GAPDH, respectively, using the 2^−DDCT^ method.

### MiRNA mimics, inhibitors, and siRNA Transfection

MiR-7-5p mimic, NC mimic, miR-7-5p inhibitors, NC inhibitors, si-AMBRA1-1, si-AMBRA1-2, c-Myc siRNA and si-NC were synthesized by Shanghai GenePharma Co., Ltd. C-MYC overexpression vector were purchased by General Biosystems (Anhui, China). Before transfection, SU-DHL-4 and SU-DHL-10 cells were seeded into six-well plates (approximately 80% confluence). Cells were transfected for 48 h prior to subsequent experiments. The transfection process was performed with Lipofectamine 3000 according to the product manuals.

### Dual-luciferase reporter gene assay

The dual-luciferase reporter gene assay was done in 293 T cells. It was implemented using the Transdetect Double-Luciferase Reporter Assay Kit (full gold # FR201-01), according to the manufacturer’s protocol. Cell lysates were utilized in the dual luciferase reporter gene assay to determine luciferase activity. Briefly, cells were transfected with mimic miR-7-5p-3′-UTR-WT or mimic miR-7-5p-3′-UTR-mut vectors and the firefly luciferase was detected once they were active. After a 24 h transfection, the cells were harvested and the luciferase signals were detected with the Dual-Luciferase Reporter Assay System. The relative activity of the two reporters were measured and calculated as ΔCT. The experiment was conducted at least 3 times.

### Apoptosis assays

For apoptosis detection, cells were collected and incubated with 5 uL Annexin V and 5 uL 7-AAD for 15 min in the dark. Analysis of apoptosis was performed using a flow cytometer (BD Biosciences Accuri C6).

### Immunofluorescence staining

SU-DHL-10 cells were transfected with si-AMBRA1 or control miRNA. The cells were fixed and stained with rabbit anti-LC3II (Invitrogen, USA). Goat anti-rabbit IgG (Invitrogen, USA) was used as the secondary antibody. Photos were taken with a fluorescence microscope (Cnoptec BK6000, China).

### In vivo experiments

To construct a tumor bearing model of the miR-7-5p overexpression lentivirus, 10 SCID mice were first injected with2.18 × 10^7^ SU-DHL-10 cells under the right armpit. When the tumor size reached 7.9 mm (tumor volume of 250 mm^3^) the mice were randomly divided into two groups (n = 5/group). Each group was treated with the miR-7-5p mimic overexpression virus or the NC overexpression virus at a volume of 6*10 ^ 8TU/mouse, and the tumors were collected 2 weeks later. Tumor volume was calculated using the formula: long diameter*short diameter*short diameter/2). The main observation indicators were (1) Changes in tumor volume: observation with the naked eye and use of vernier calipers to measure tumor length and width once a week. The weight of the mice was measured weekly, (2) Tumor volume and weight at end point: after 14 days, the mice were killed and tumors were dissected; tumor volume was measured and weighed as above. (3) Treatment of tumor specimens: half of the dissected tumors were fixed with 4% paraformaldehyde and half were frozen in liquid nitrogen and stored at − 80 °C.

### TUNEL staining

Apoptosis was assessed via TUNEL staining. Briefly, tumor tissues were fixed with 4% formaldehyde (Thermo Fisher Scientific) and then stained using the TUNEL Apoptosis Detection Kit III, FITC (Boster, Wuhan, China). Upon analysis, cells positive for TUNEL staining were visualized and counted under a fluorescence microscope (Olympus IX73, Japan).

### Immunohistochemistry

The mouse tissue sections were deparaffinized following standard procedures. Then, the antigens were unmasked, and the sections were incubated with an anti-Ki-67 (rabbit, Affinity, China) and an appropriate secondary antibody. Images were captured using a Digital Pathology System (Pannoramic MIDI, 3DHISTECH).

### Western blotting

Antibodies against AMBRA1 and c-MYC were purchased from Abcam and the LC3 antibody was purchased from Sigma. Antibodies against p-c-MYC, mTOR, PP2A-C, PARP, and p-cmyc were purchased from Cell Signaling Technology. Other antibodies such as p62 and GAPDH were purchased from Proteintech. Please refer to Supplementary Table 3 for detailed catalog numbers and dilutions of the antibodies. Total proteins were extracted from the cell lines and equal amounts of protein were separated by electrophoresis and then transferred onto PVDF membranes. The membranes were blocked with 5% nonfat dried milk powder in TBST and incubated with primary antibodies against target proteins and GAPDH at 4 °C overnight. Then, membranes were washed and incubated with horseradish peroxidase-conjugated secondary antibodies for 2 h at room temperature. The protein bands were visualized using an enhanced chemiluminescence kit; GAPDH was used as the internal control. Quantitative data were analyzed using ImageJ software (NIH).

### Statistical analysis

Data are presented as the mean ± SD. SPSS 26.0 statistical software and GraphPad Prism 7.0 were used for analysis. Multiple groups of data were analyzed by one-way ANOVA, and two groups of data were analyzed by Student’s *t*-test. *P* < 0.05 was considered significant.

## Results

### Upregulation of miR‑7‑5p and downregulation of AMBRA1 in DLBCL cell lines

**Fig. 1:**
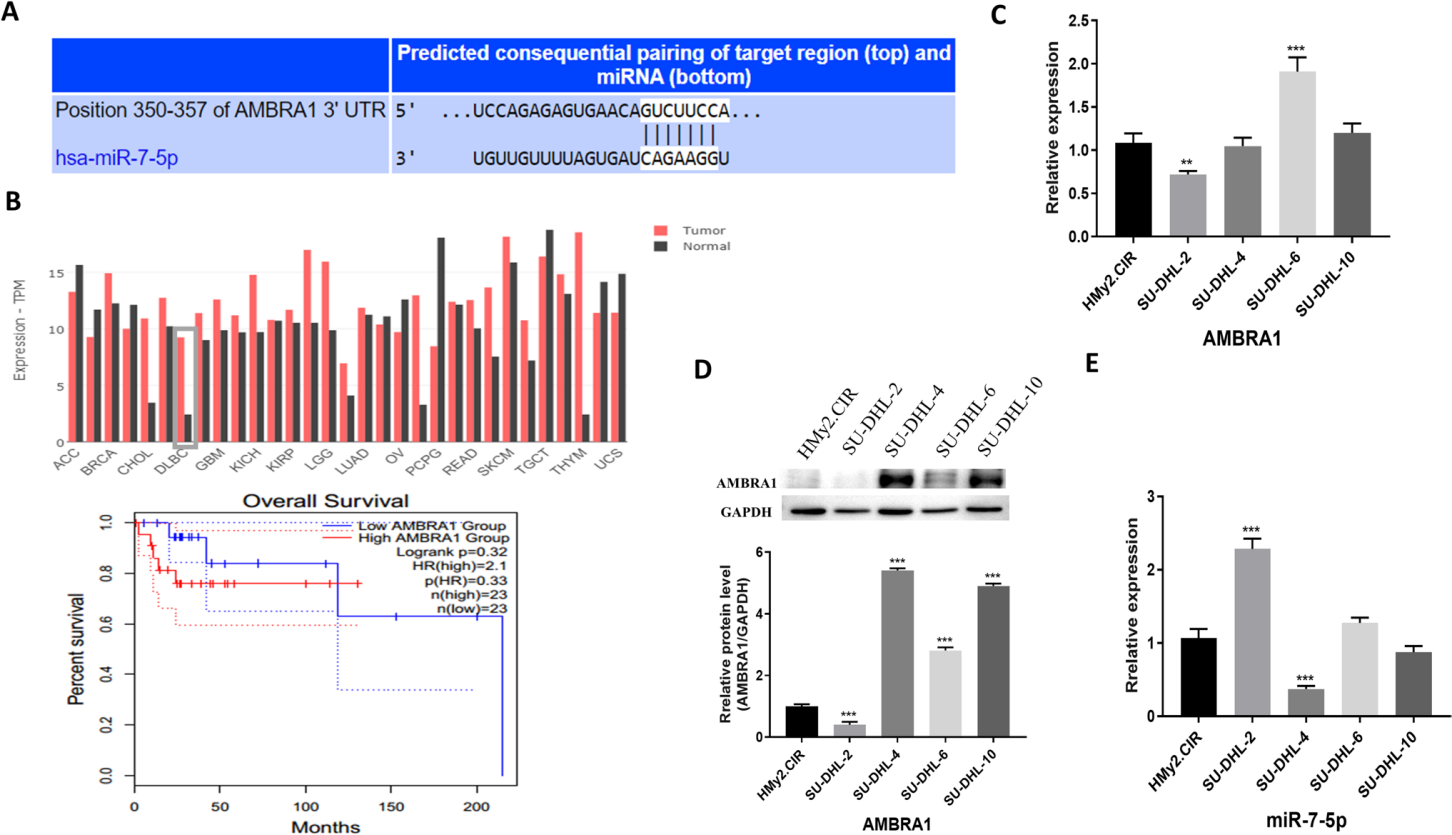
MiR-7-5p was upregulated in DLBCL tissues. **A** Screening of target genes of miR-7-5p through the TargetScan database. **B** The level of AMBRA1 in DLBCL tissues and adjacent non-tumor tissues identified with the TCGA database. **C** and **D** AMBRA1 expression in human B lymphoblast HMy2.CIR cells and the human DLBCL cell lines SU-DHL-2, SU-DHL-4, SU-DHL-6, and SU-DHL-10. **E** MiR-7-5p expression in human B lymphoblast HMy2.CIR cells and the human DLBCL cell lines SU-DHL-2, SU-DHL-4, SU-DHL-6, and SU-DHL-10. Quantitative data are presented as mean ± SD. ***p < 0.001 compared with the control group. AMBRA1, activating molecule in Beclin-1-regulated autophagy

Using the TargetScan database (http://www.targetscan.org/vert_71/), we identified AMBRA1 as a target gene of miR‑7‑5p (Fig. 1A). Using the TCGA database, we found that AMBRA1 exhibited different expression levels in different tumors compared with normal tissues. AMBRA1 expression was elevated in DLBCL. However, no difference in the overall survival of patients with high expression of AMBRA1 versus those with low expression of AMBRA1 was observed (Fig. 1B). To better understand the regulatory process of miR-7-5p in the pathogenesis of DLBCL, we first determined the expression of miR-7-5p and AMBRA1 in DLBCL cell lines. WB results showed that the expression of AMBRA1 in SU-DHL-4 and SU-DHL-10 cells was higher than in normal cells (Fig. 1C, D). In addition, qPCR results showed that expression of miR-7-5p was decreased in SU-DHL-4 and SU-DHL-10 cells (Fig. 1E). Therefore, SU-DHL-4 and SU-DHL-10 cells were selected for subsequent cell experiments.

### AMBRA1 is a direct target of miR‑7‑5p in DLBCL

**Fig. 2:**
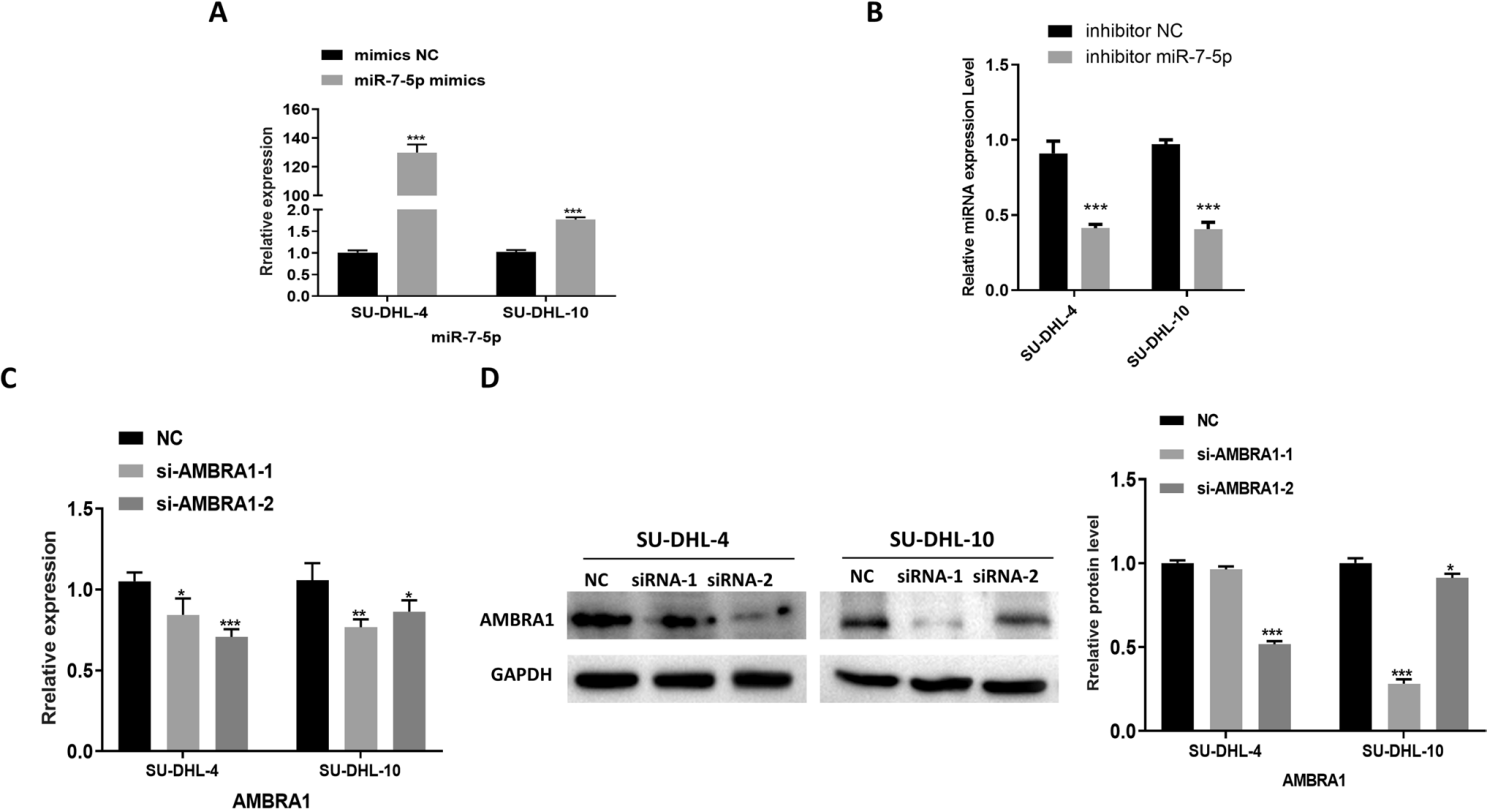
Construction and transfection of the miR-7-5p inhibitor, miR-7-5p mimics, and AMBRA1 siRNA. **A** and **B** qPCR assay confirming the transfection efficiency of the miR-7-5p mimic and inhibitor. **C** and **D** qPCR and western blot assays confirming the transfection efficiency of si-AMBRA1. The data are presented as mean ± SD. NC, negative control. ***p < 0.001 compared with the NC group

To further confirm the regulatory effect of miR-7-5p and AMBRA1 on DLBCL cells, miR-7-5p inhibitor, miR-7-5p mimics, and AMBRA1 siRNA were used. We found that compared with the control, the expression of miR-7-5p was increased in SU-DHL-4 and SU-DHL-10 cells with the miR-7-5p mimics. The effect of the miR-7-5p inhibitor was opposite to that of the miR-7-5p mimics (Fig. 2A, B). The expression of AMBRA1 in the SU-DHL-4 and SU-DHL-10 cell lines with siAMBRA1-1 and siAMBRA1-2 was lower than in the control (Fig. 2C, D). However, in the siAMBRA1-2 group, the inhibition was more pronounced. Therefore the following experiments were conducted with si-AMBRA1-2.

**Fig. 3:**
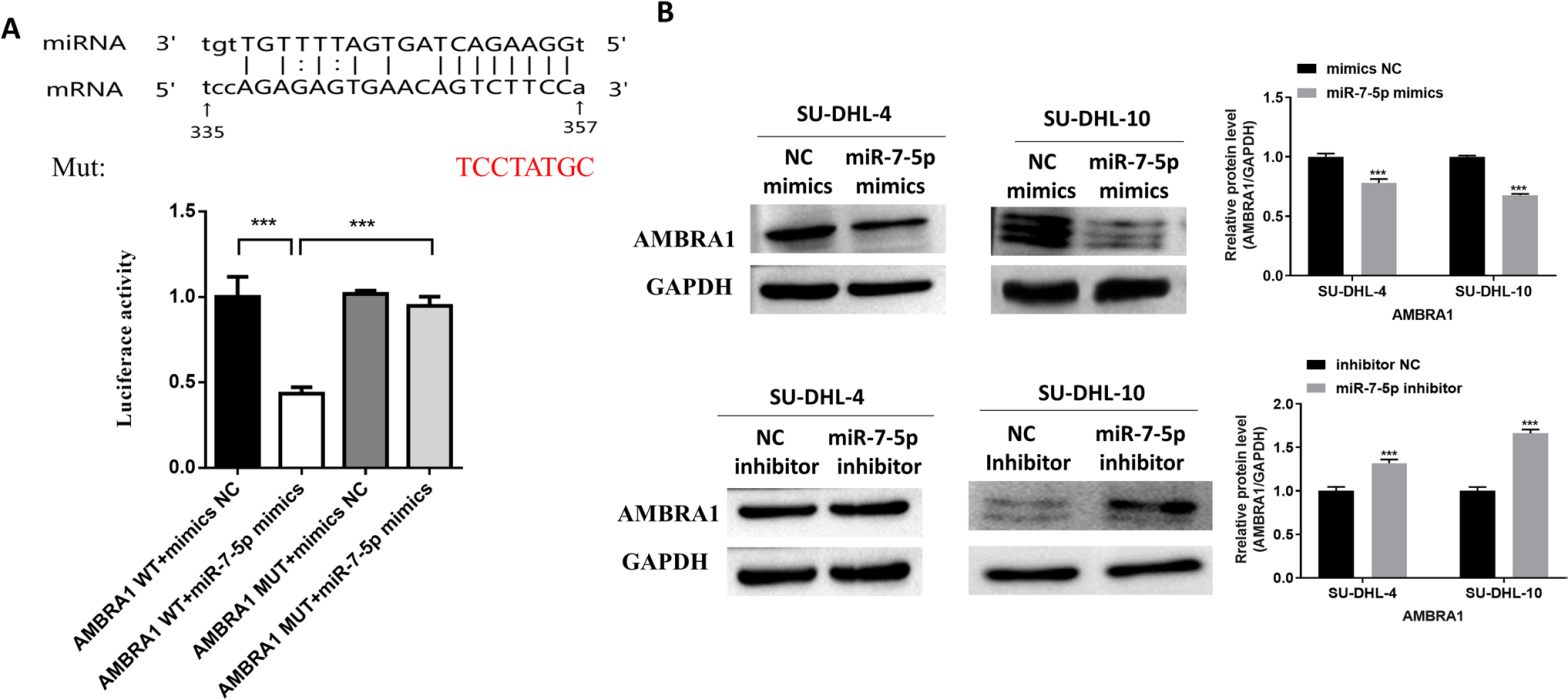
MiR‑7-5p directly targets AMBRA1 in DLBCL cells. **A** Luciferase reporter assay and the binding sites of wild-type of AMBRA1 3’‑UTR combined with miR‑7‑5p. **B** The protein expression of AMBRA1 was analyzed in cells containing miR‑7-5p mimic or inhibitor. Quantitative data are presented as the means ± SD. NC, negative control, ***p < 0.001 vs. NC

Luciferase reporter assays were carried out to verify that miR‑7‑5p directly targets AMBRA1. Through analysis of the TargetScan database, we found that the binding sequence of miR‑7‑5p and AMBRA1sequence was AGAGAGTGAACAGTCTTCCA. Then, we mutated the seed region sequence from AGTCTTCC to TCCTATGC. Interestingly, after mutation of the binding site, fluorescent activity of the cells containing the miR‑7‑5p mimic and the mutant AMBRA1 increased significantly. However, miR-7-5p maintained its inhibitory effect upon transfection of AMBRA1 containing the wild type seed region (Fig. 3A). These results identified AMBRA1 as a direct target of miR-7-5p.

AMBRA1 expression in SU-DHL-4 and SU-DHL-10 cells containing miR‑7‑5p mimics or inhibitor was also detected. Results showed that compared to controls, the protein expression level of AMBRA1 was significantly reduced in SU-DHL-4 and SU-DHL-10 cells with miR‑7‑5p mimics and increased in cells containing the miR‑7‑5p inhibitor (Fig. 3B). In brief, miR‑7‑5p regulated AMBRA1 expression in DLBCL.

### MiR-7-5p regulated the AMBRA1 signaling pathway in DLBCL

**Fig. 4:**
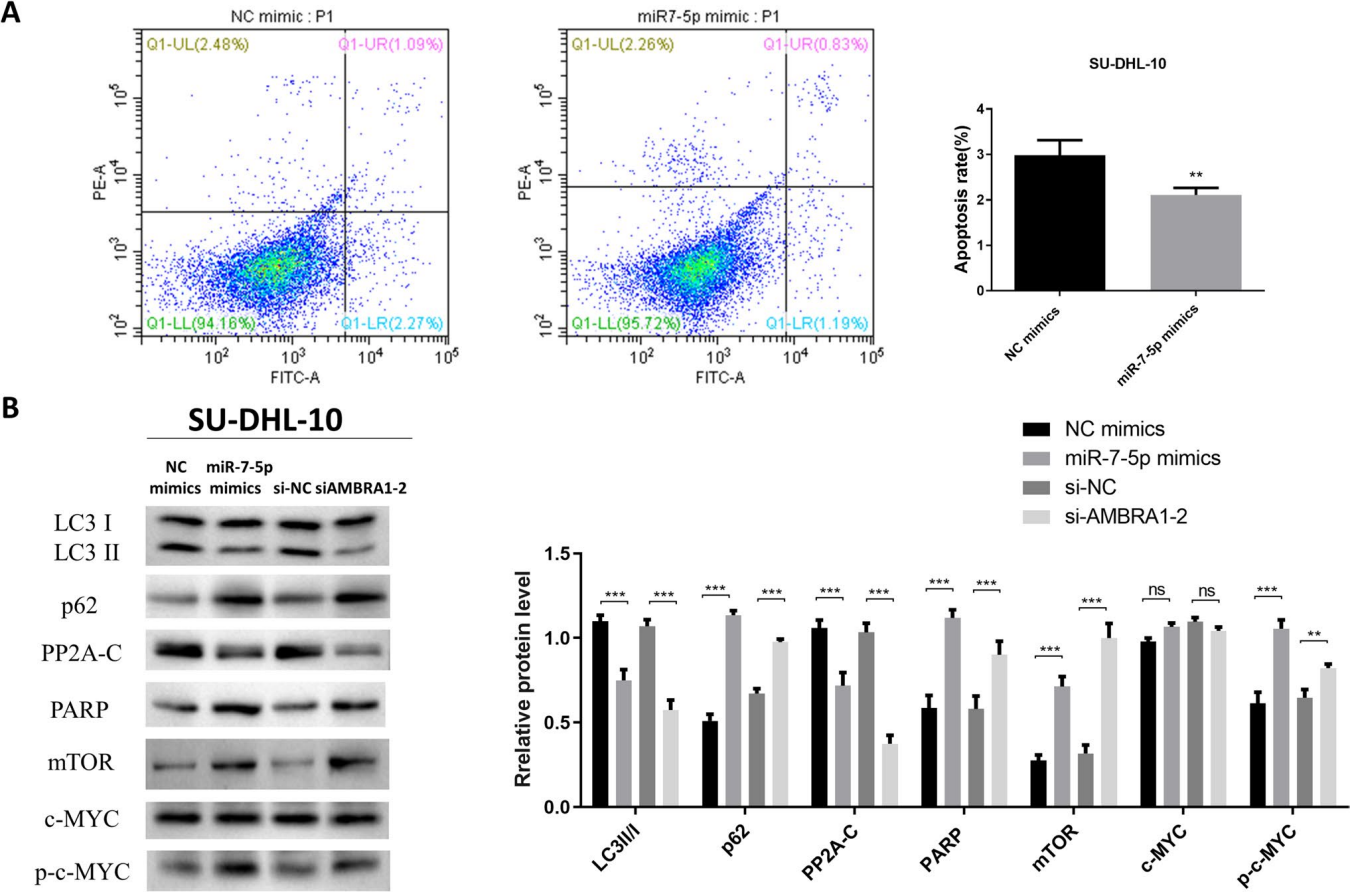
MiR-7-5p suppresses DLBCL cells autophagy and apoptosis. **A** Cell apoptosis was examined using flow cytometry. **B** The protein levels of mTOR, c-MYC, autophagy and apoptosis proteins transfected with miR-7-5p mimic or si-AMBRA1 by western blot assays. Data were shown as mean ± SD. ***p < 0.001 compared with the NC group. NS indicated no significance

Next, flow cytometry was performed to measure apoptosis in SUDHL-4 and SU-DHL-10 cells. Results showed that compared with the NC group, the percentage of apoptotic cells in the miR-7-5p overexpression group was decreased (Figs. 4A and S1). Western blotting was used to measure the expression level of apoptosis- and autophagy-related proteins. Results showed that in SU-DHL-10 cells, PP2A-C and LC3II were down regulated by miR-7-5p overexpression and silencing of AMBRA1, where p-c-MYC, mTOR, and p62 were upregulated (Fig. 4B). Western blotting experiments in SU-DHL-4 cells also confirmed that overexpression of miR-7-5p down regulated LC3II, where PARP and p62 were upregulated (Fig. S1). The downregulation of LC3II and the upregulation of p62 indicated that autophagy was decreased. Further, an increase in the PARP protein suggested that the inhibition of AMBRA1 may decrease activation of the apoptosis pathway. We found that AMBRA1 enhanced PP2A-C activity upon c-MYC dephosphorylation. Additionally, we found that PP2A directly dephosphorylated c-MYC in fibroblast REF52 cells, thereby destabilizing c-MYC [29]. Moreover, this AMBRA1 and PP2A-mediated regulation of c-MYC was controlled by mTOR [21]. Therefore, inhibition of AMBRA1 led to an increase in mTOR, a decrease in PP2A-C, and an increase in c-MYC reactivity, which is not conducive to apoptosis. The above results revealed that miR-7-5p overexpression and silencing of AMBRA1 inhibited autophagy in DLBCL cells. In addition, the flow cytometry and WB results showed that the overall effect of AMBRA1 silencing was to decrease the occurrence of apoptosis, which mainly relies on the AMBRA1/c-MYC pathway.

**Fig. 5:**
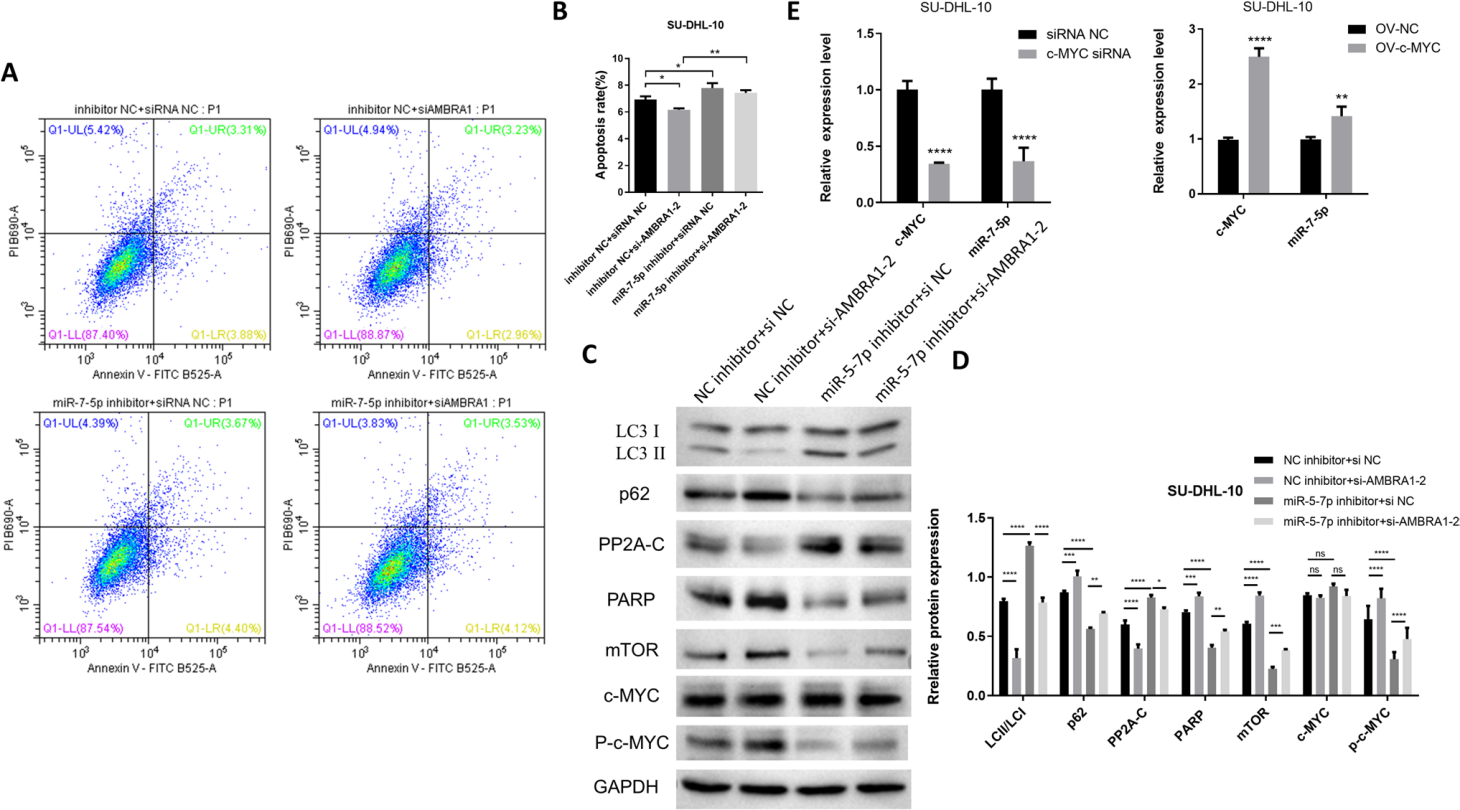
MiR-7-5p regulated DLBCL autophagy and apoptosis in vitro by targeting AMBRA1. **A**, **B** Cell apoptosis was examined using flow cytometry. **C**, **D** The protein levels of mTOR, c-MYC, autophagy and apoptosis proteins transfected with miR-7-5p inhibitor and si-AMBRA1 by western blot assays. **E** Detection of positive feedback relationship between c-MYC and miR-7-5p by qPCR. Data were shown as mean ± SD. NC, negative control, ns indicated no significance, ***p < 0.001 vs. NC

We then carried out rescue experiments in SU-DHL-10 cells. Flow cytometric analysis showed that si-AMBRA1 decreased the apoptosis promoting effect of the miR-7-5p inhibitor (Fig. 5A, B). WB results showed that silencing miR-7-5p increased PP2A-C and LC3II expression, where PARP, p-c-MYC, mTOR, and p62 were down regulated. In contrast, silencing AMBRA1 had the opposite effects. In the combination group treated with the miR-5-7p inhibitor and si-AMBRA1, we found that si-AMBRA1 inhibited the apoptosis and autophagy promoting effect of the miR-7-5p inhibitor (Fig. 5C, D). The above results demonstrate that miR-7-5p regulated autophagy and apoptosis in DLBCL cells through AMBRA1.

An interesting finding was that c-MYC had a feedback effect on miR-7-5p expression. Silencing of c-MYC led to a decrease in miR-7-5p, while overexpression of c-MYC caused an increase in miR-7-5p (Fig. 5E). This result suggests that in DLBCL, the expression of miR-7-5p is also regulated the downstream c-MYC, which can promote the transcription of miR-7-5p. Moreover, blocking the expression of miR-7-5p in SU-DHL-10 cells resulted in an increase in AMBRA1 and a decrease in p-c-MYC levels. This revealed a positive feedback loop of the miR-7-5p- AMBRA1-MYC axis in DLBCL.

### Autophagy inhibitors increased apoptosis by inhibiting the AMBRA1 autophagy pathway

**Fig. 6:**
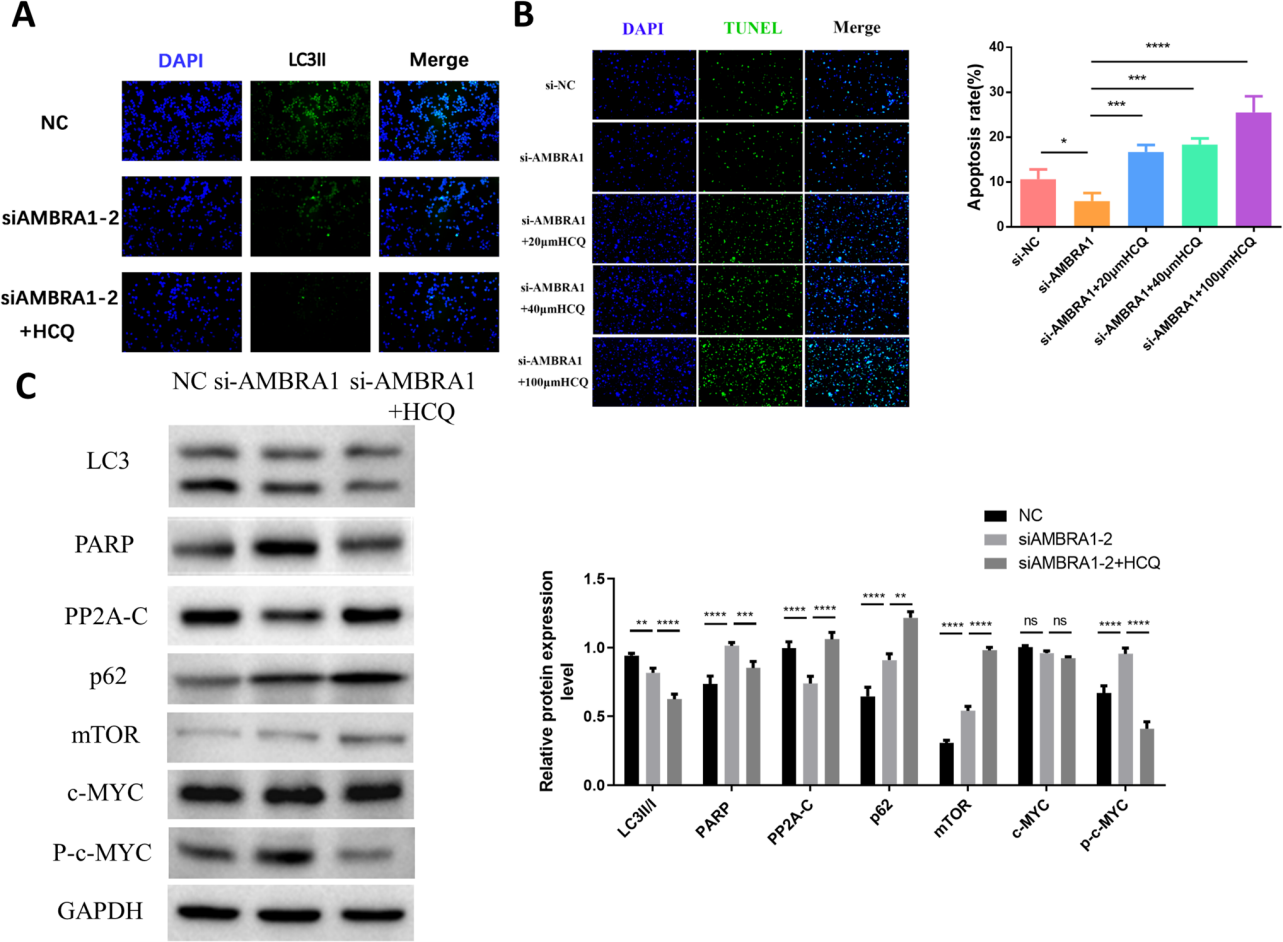
Addition of autophagy inhibitor promotes DLBCL cell apoptosis. **A** Addition of HCQ to immunofluorescence reduces autophagy. **B** Cell apoptosis was examined using tunnel test. **C** The protein levels of mTOR, c-MYC, autophagy and apoptosis proteins transfected with si-AMBRA1 or si-AMBRA1 + HCQ by western blot assays. Data were shown as mean ± SD. *NC* negative control, ***p < 0.001 vs. NC. HCQ, Hydroxychloroquine

AMBRA1 is at the intersection of apoptosis and autophagy. However, it is unknown if inhibition of autophagy results in an increase apoptosis. Therefore, we inhibited autophagy via HCQ and observed the combined effect of HCQ and AMBRA1. Immunofluorescence experiments showed that compared with the NC group, LC3II expression was decreased after AMBRA1 silencing and the addition of HCQ further reduced its expression (Fig. 6A). Through TUNEL staining, we also determined that the optimal HCQ concentration was 100 μm (Fig. 6B). WB experiments showed that the addition of HCQ after AMBRA1 silencing further changed the expression of autophagic proteins (LC3II/I and p62). However, the expression of apoptotic proteins such as PP2A-C increased after the addition of HCQ, indicating that inhibition of autophagy can feed back and activate the AMBRA1-related apoptotic pathway (Fig. 6C).

### Overexpression of miR-7-5p reduced autophagy and apoptosis in vivo

**Fig. 7:**
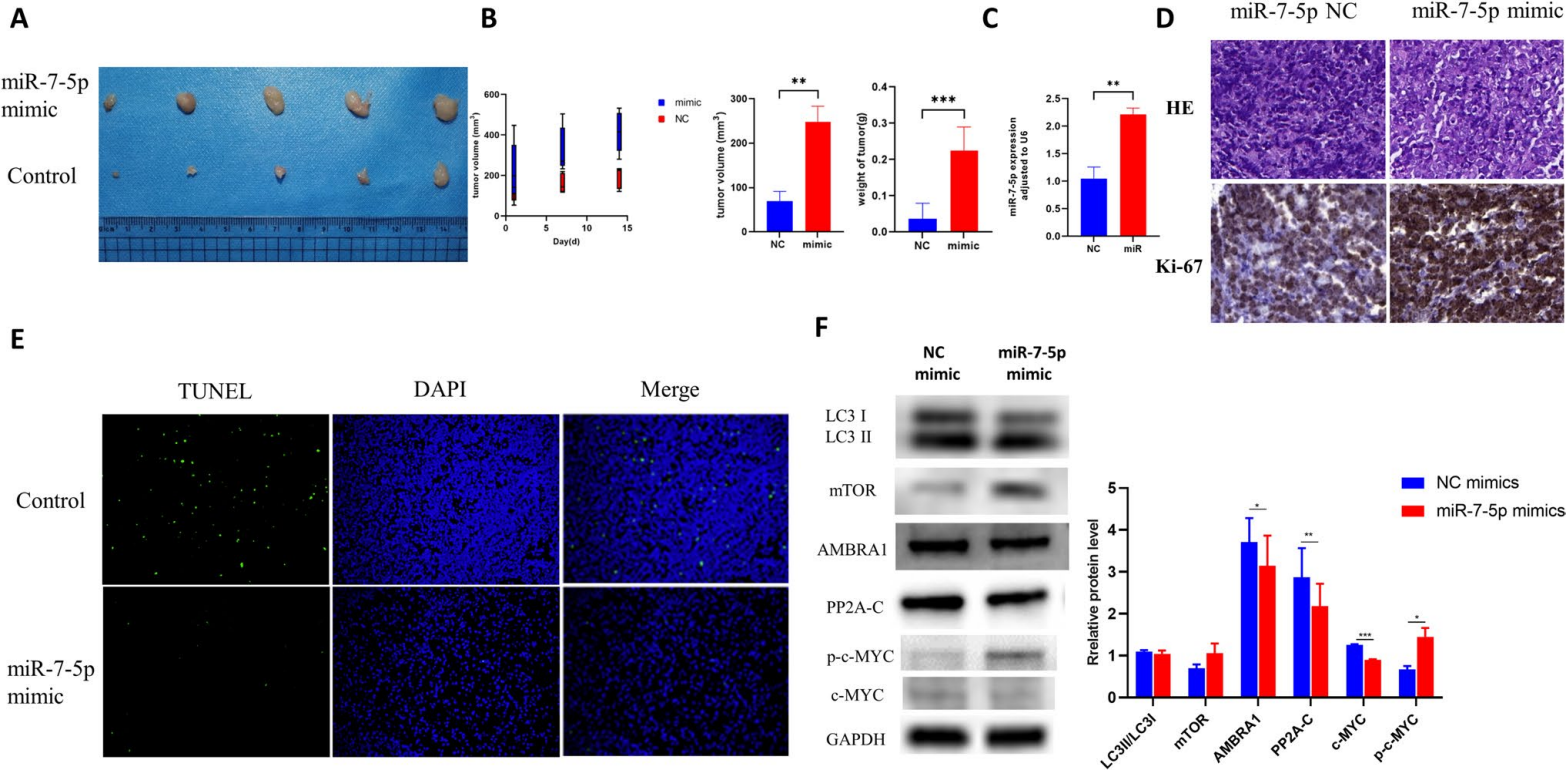
Overexpression of miR-7-5p accelerates tumor growth in vivo. The SCID mouse xenograft was established by the intratumoral injection of miR-7-5p mimic. **A** The tumor images in the miR-7-5p mimic-NC group (*n* = 5) and in the miR-7-5p mimic group (*n* = 5). **B** The tumor volume and weight of tumor in the miR-7-5p mimic-NC group (*n* = 5) and in the miR-7-5p mimic group (*n* = 5). **C** The relative RNA levels of miR-7-5p were evaluated by qPCR between miR-7-5p mimic and miR-7-5p mimic-NC of tumor tissues. **D** Representative images of HE staining and Immunohistochemistry of tumor issues in different groups (the higher scale bar indicates 100 µm). **E** Representative images of tunel staining of tumor issues in different groups (the higher scale bar indicates 100 µm). **F** The protein levels of mTOR, c-MYC, autophagy and apoptosis proteins transfected with miR-7-5p mimic or miR-7-5p mimic-NC by western blot assays. Data were shown as mean ± SD. ***p < 0.001 vs. NC. *NC* negative control

**Fig. 8:**
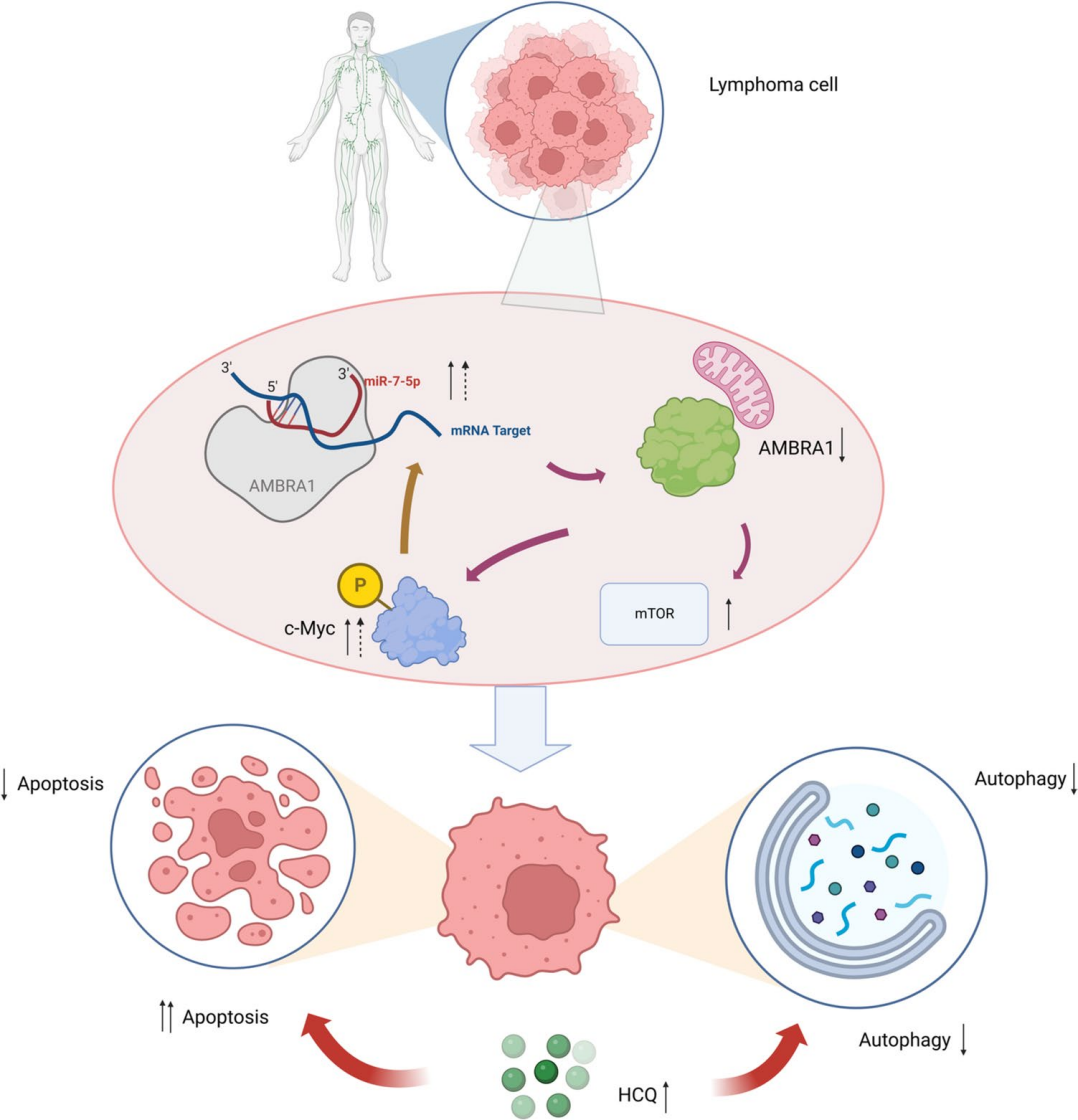
Complete chain of mechanism hypothesis. The schematic diagram illustrates the role of miR-7-5p targeting AMBRA1 in regulating autophagy and apoptosis phenotype. In addition, if autophagy inhibitor is added, miR-7-5p-mediated AMBRA1 will turn from autophagy to more apoptosis

To investigate the effects of miR-7-5p on tumorigenesis in vivo, a tumor-bearing model of miR-7-5p overexpression was constructed via intratumoral injection of miR-7-5p mimics in SCID mice. As shown in Fig. 7A and B, the miR7-5p mimic markedly increased tumor volume and weight. QPCR results showed that the expression of miR-7-5p in miR-7-5p mimic tumors was higher than in the control group (Fig. 7C). In addition, immunohistochemistry demonstrated that Ki-67 expression in the miR-7-5p mimic group was higher than in the control group (Fig. 7D). TUNEL experiments also revealed that apoptosis levels in the miR-7-5p mimic group were lower than control group (Fig. 7E). Moreover, the miR-7-5p mimic group also exhibited reduced expression of the PP2A-C, LC3II, and AMBRA1 proteins, and tended to exhibit an increase in p-c-MYC and mTOR (Fig. 7F). These data indicate that miR-7-5p mainly affected autophagy and apoptosis in *vivo* through the AMBRA1 pathway (Fig. 8).

## Discussion

This study demonstrated that miR-7-5p suppressed DLBCL autophagy and apoptosis by targeting AMBRA1 in DLBCL cells. There is increasing evidence that in cancer, post-transcriptional and post-translational control mediated by noncoding miRNA plays a significant role in autophagy. Deregulation of miRNAs has been shown to be related to the development and progression of cancer [30]. Jegga et al. proposed that miR-130, miR-98, miR-124, miR-204, and miR-142 play potential regulatory functions in the autophagic process [31]. Research findings support the view that autophagy regulation by miRNAs may be a feasible target for the treatment of cancer, as this could be used to reduce resistance to tumor hereditary hypoxia, or increase tumor cell vulnerability to chemotherapeutic drugs [32]. MiR-7-5p is regarded as a tumor suppressor in many types of cancer and has been shown to suppress cancer cell growth, proliferation, migration, and invasion, as well as increasing sensitivity of resistant tumor cells to therapy. On the contrary, some studies have also identified an oncogenic role of miR-7-5p in certain cancer types. For example, studies demonstrated that miR-7-5p inhibited EGFR mRNA and protein expression and downstream AKT and ERK1/2 activity in head and neck [33], breast, lung, and prostate cancers [34], as well as in glioblastoma multiforme [35]. Chou et al. showed that the inhibition of miR-7-5p expression suppressed the growth of lung carcinoma [36]. In addition, Yu et al. found that the expression of miR-7 was upregulated in renal cell carcinoma compared with normal tissues, and the downregulation of miR-7 resulted in reduced cell migration and proliferation and induced apoptosis [12, 37]. At present, no studies have clarified the role of miR-7-5p in DLBCL. Compared with the different effects of miR-7-5p on other cancers, we found that miR-7-5p plays a dual role in DLBCL by inhibiting both autophagy and apoptosis. These effects are closely related to the regulation of AMBRA1 by miR-7-5p.

AMBRA1 is a positive regulator of the autophagy pathway, affecting cell proliferation in vitro and in vivo [21, 38]. AMBRA1 also plays a role upstream of mTORC1-dependent autophagy by stabilizing the kinase ULK1 (UNC-51 like autophagy activated kinase 1) and promoting formation of the autophagosome core complex [38]. AMBRA1 is also an important target of apoptotic protease, leading to inhibition of the autophagy mechanism and completion of the cell death program. Pagliarini et al. found that in the early stage of apoptosis, degradation of the AMBRA1 protein by caspases and calpains aids in the elimination of the pro-survival function of autophagy [39]. AMBRA1 is reported to be an important regulator of autophagy and apoptosis in ovarian cancer cells under the influence of cisplatin, which can maintain the balance between autophagy and apoptosis [19]. In this study, we also demonstrated a close regulatory effect of AMBRA1 on autophagy and apoptosis in DLBCL, and this effect was regulated by the upstream regulator miR-7-5p.

Our research found that when miR-7-5p and AMBRA1 changed, the proteins related to apoptosis, including c-MYC and PP2A-C, also changed. In most lung cancer cell lines, low expression levels of AMBRA1 and high expression levels of phosphorylated MYC have been reported [21]. Our study found that in DLBCL, miR-7-5p prevented MYC dephosphorylation through AMBRA1 downregulation, which is consistent with the lung cancer research. These data suggest that miR-7-5p is a novel miRNA in DLBCL, and may affect tumorigenesis by regulating the AMBRA1 pathway [38].

More interestingly, we found that when the AMBRA1-mediated autophagy effect was blocked by hydroxychloroquine, AMBRA1 promoted apoptosis instead of autophagy. The autophagy inhibitor hydroxychloroquine plays an important role in anti-tumor therapy due to its effect on autophagy. Other anti-tumor mechanisms of hydroxychloroquine include induction of cell death through apoptosis and necrosis, and immune regulation/anti-inflammatory activities [40]. Previous studies found that targeted knockout of the autophagy-related protein ATG7 or use of the autophagy inhibitor 3-methyladenine inhibited caspase activation and reduced apoptosis [41]. However, these results are contrary to the results of our study. The difference may be due to the regulation of the AMRBA1 gene. When a gene is involved in more than two pathways, it is possible to translate the activation of one pathway into another. This phenomenon may have a positive effect on anti-tumor activities. It is worth noting that we discovered a regulatory loop involving mutual regulation between miR-7-5p and c-MYC. On the one hand, miR-7-5p directly downregulated AMBRA1 and upregulated c-MYC expression after transcription. On the other hand, the increase in c-MYC further upregulated the transcription of miR-7-5p, leading to a reduction in AMBRA1 expression. Blockade of autophagy may further promote the production of apoptosis, and is therefore a potential target for future DLBCL treatments.

While we have elucidated the oncogenic role of miR-7-5p and its molecular mechanisms in DLBCL both in vitro and in vivo, there are still some limitations that need to be discussed. Firstly, it is necessary to assess the levels of miR-7-5p in clinical DLBCL samples for further validation additionally, while we report here that the addition of the autophagy inhibitor HCQ promotes the shift of the AMBRA1-regulated pathway towards apoptosis, further experiments are required to determine if similar effects can be achieved by inhibiting autophagy through alternative means.

## Conclusions

Our findings support miR-7-5p as a promising biomarker and therapeutic target in DLBCL. We demonstrated that miR-7-5p induced autophagy and apoptosis by regulating AMBRA1 in DLBCL cells. Inhibition of autophagy regulated the transition of the AMBRA1-mediated pathway in DLBCL to an apoptotic phenotype. Through the miR-7-5p and c-MYC regulatory loop, we believe that this interaction may be crucial to the occurrence and development of DLBCL.

## Supplementary Information

Below is the link to the electronic supplementary material.

Supplementary file1 (DOCX 18 KB)

Supplementary file2 (DOCX 15 KB)

Supplementary file3 (DOCX 16 KB)

Figure S1. MiR-7-5p suppresses SUDHL-4 cells autophagy and apoptosis. (A) The protein levels of autophagy and apoptosis proteins transfected with miR-7-5p mimics by western blot assays. (B) Cell apoptosis was examined using flow cytometry. Data were shown as mean ± SD. ***p < 0.001 compared with the NC group. NS indicated no significance. Supplementary file4 (TIF 252 KB)

## Data Availability

All data and material during the current study are available from the corresponding author on reasonable request.

## References

[1] Chihara D, Nastoupil LJ, Williams JN, Lee P, Koff JL, Flowers CR (2015) New insights into the epidemiology of non-Hodgkin lymphoma and implications for therapy. Expert Rev Anticancer Ther 15(5):531–544 25864967 10.1586/14737140.2015.1023712 PMC4698971

[2] Liu Y, Barta SK (2019) Diffuse large B-cell lymphoma: 2019 update on diagnosis, risk stratification, and treatment. Am J Hematol 94(5):604–616 30859597 10.1002/ajh.25460

[3] The non-Hodgkin’s lymphoma classification project (1997) A clinical evaluation of the international lymphoma study group classification of non-Hodgkin’s lymphoma. *Blood* 89(11): 3909–3918 9166827

[4] Sabattini E, Bacci F, Sagramoso C, Pileri SA (2010) WHO classification of tumours of haematopoietic and lymphoid tissues in 2008: an overview. Pathologica 102(3):83–87 21171509

[5] Flowers CR, Sinha R, Vose JM (2010) Improving outcomes for patients with diffuse large B-cell lymphoma. CA: Cancer J Clin 60(6):393–408 21030533 10.3322/caac.20087

[6] Niitsu N, Okamoto M, Nakamura N, Nakamine H, Bessho M, Hirano M (2007) Clinicopathologic correlations of stage IE/IIE primary thyroid diffuse large B-cell lymphoma. Ann Oncol: Off J Eur Soc Med Oncol 18(7):1203–1208 10.1093/annonc/mdm094 17429099

[7] Xuefang Z, Ruinian Z, Liji J, Chun Z, Qiaolan Z, Jun J, Yuming C, Junrong H (2020) miR-331-3p inhibits proliferation and promotes apoptosis of nasopharyngeal carcinoma cells by targeting elf4B-PI3K-AKT pathway. Technol Cancer Res Treat 19:1533033819892251 31984860 10.1177/1533033819892251 PMC6985969

[8] Ali Syeda Z, Langden SSS, Munkhzul C, Lee M, Song SJ (2020) Regulatory mechanism of MicroRNA expression in cancer. Int J Mol Sci 21(5):1723 32138313 10.3390/ijms21051723 PMC7084905

[9] Cimmino A, Calin GA, Fabbri M, Iorio MV, Ferracin M, Shimizu M, Wojcik SE, Aqeilan RI, Zupo S, Dono M et al (2005) miR-15 and miR-16 induce apoptosis by targeting BCL2. Proc Natl Acad Sci USA 102(39):13944–13949 16166262 10.1073/pnas.0506654102 PMC1236577

[10] Xu Y, Liu Z, Lv L, Li P, Xiu B, Qian W, Liang A (2020) MiRNA-340-5p mediates the functional and infiltrative promotion of tumor-infiltrating CD8(+) T lymphocytes in human diffuse large B cell lymphoma. J Exp Clin Cancer Res 39(1):238 33168024 10.1186/s13046-020-01752-2 PMC7653890

[11] Arribas AJ, Campos-Martin Y, Gomez-Abad C, Algara P, Sanchez-Beato M, Rodriguez-Pinilla MS, Montes-Moreno S, Martinez N, Alves-Ferreira J, Piris MA et al (2012) Nodal marginal zone lymphoma: gene expression and miRNA profiling identify diagnostic markers and potential therapeutic targets. Blood 119(3):e9–e21 22110251 10.1182/blood-2011-02-339556

[12] Kalinowski FC, Brown RA, Ganda C, Giles KM, Epis MR, Horsham J, Leedman PJ (2014) microRNA-7: a tumor suppressor miRNA with therapeutic potential. Int J Biochem Cell Biol 54:312–317 24907395 10.1016/j.biocel.2014.05.040

[13] Shi Y, Luo X, Li P, Tan J, Wang X, Xiang T, Ren G (2015) miR-7-5p suppresses cell proliferation and induces apoptosis of breast cancer cells mainly by targeting REGγ. Cancer Lett 358(1):27–36 25511742 10.1016/j.canlet.2014.12.014

[14] Giles KM, Brown RA, Epis MR, Kalinowski FC, Leedman PJ (2013) miRNA-7-5p inhibits melanoma cell migration and invasion. Biochem Biophys Res Commun 430(2):706–710 23206698 10.1016/j.bbrc.2012.11.086

[15] Ma C, Qi Y, Shao L, Liu M, Li X, Tang H (2013) Downregulation of miR-7 upregulates Cullin 5 (CUL5) to facilitate G1/S transition in human hepatocellular carcinoma cells. IUBMB Life 65(12):1026–1034 24339204 10.1002/iub.1231

[16] Liu Z, Liu Y, Li L, Xu Z, Bi B, Wang Y, Li JY (2014) MiR-7-5p is frequently downregulated in glioblastoma microvasculature and inhibits vascular endothelial cell proliferation by targeting RAF1. Tumour Biol: J Int Soc Oncodev Biol Med 35(10):10177–10184 10.1007/s13277-014-2318-x 25027403

[17] Li Q, Wu X, Guo L, Shi J, Li J (2019) MicroRNA-7-5p induces cell growth inhibition, cell cycle arrest and apoptosis by targeting PAK2 in non-small cell lung cancer. FEBS Open Bio 9(11):1983–1993 31587474 10.1002/2211-5463.12738 PMC6823280

[18] Strappazzon F, Vietri-Rudan M, Campello S, Nazio F, Florenzano F, Fimia GM, Piacentini M, Levine B, Cecconi F (2011) Mitochondrial BCL-2 inhibits AMBRA1-induced autophagy. EMBO J 30(7):1195–1208 21358617 10.1038/emboj.2011.49 PMC3094111

[19] Li X, Zhang L, Yu L, Wei W, Lin X, Hou X, Tian Y (2016) shRNA-mediated AMBRA1 knockdown reduces the cisplatin-induced autophagy and sensitizes ovarian cancer cells to cisplatin. J Toxicol Sci 41(1):45–53 26763392 10.2131/jts.41.45

[20] Fimia GM, Di Bartolomeo S, Piacentini M, Cecconi F (2011) Unleashing the Ambra1-Beclin 1 complex from dynein chains: Ulk1 sets Ambra1 free to induce autophagy. Autophagy 7(1):115–117 21079415 10.4161/auto.7.1.14071

[21] Cianfanelli V, Fuoco C, Lorente M, Salazar M, Quondamatteo F, Gherardini PF, De Zio D, Nazio F, Antonioli M, D’Orazio M et al (2015) AMBRA1 links autophagy to cell proliferation and tumorigenesis by promoting c-Myc dephosphorylation and degradation. Nat Cell Biol 17(1):20–30 25438055 10.1038/ncb3072 PMC4976803

[22] Nazio F, Strappazzon F, Antonioli M, Bielli P, Cianfanelli V, Bordi M, Gretzmeier C, Dengjel J, Piacentini M, Fimia GM et al (2013) mTOR inhibits autophagy by controlling ULK1 ubiquitylation, self-association and function through AMBRA1 and TRAF6. Nat Cell Biol 15(4):406–416 23524951 10.1038/ncb2708

[23] Kang R, Zeh HJ, Lotze MT, Tang D (2011) The Beclin 1 network regulates autophagy and apoptosis. Cell Death Differ 18(4):571–580 21311563 10.1038/cdd.2010.191 PMC3131912

[24] Fimia GM, Corazzari M, Antonioli M, Piacentini M (2013) Ambra1 at the crossroad between autophagy and cell death. Oncogene 32(28):3311–3318 23069654 10.1038/onc.2012.455

[25] Liu J, Chen Z, Guo J, Wang L, Liu X (2019) Ambra1 induces autophagy and desensitizes human prostate cancer cells to cisplatin. Biosci Rep. 10.1042/BSR20170770 10.1042/BSR20170770 PMC6706594 29101240

[26] Sun WL, Wang L, Luo J, Zhu HW, Cai ZW (2019) Ambra1 inhibits paclitaxel-induced apoptosis in breast cancer cells by modulating the Bim/mitochondrial pathway. Neoplasma 66(3):377–385 30784282 10.4149/neo_2018_180710N467

[27] Cianfanelli V, D’Orazio M, Cecconi F (2015) AMBRA1 and BECLIN 1 interplay in the crosstalk between autophagy and cell proliferation. Cell Cycle (Georgetown, Tex) 14(7):959–963 25803737 10.1080/15384101.2015.1021526 PMC4615147

[28] Cianfanelli V, Cecconi F (2015) AMBRA1: when autophagy meets cell proliferation. Autophagy 11(9):1705–1707 26101901 10.1080/15548627.2015.1053681 PMC4590640

[29] Arroyo JD, Hahn WC (2005) Involvement of PP2A in viral and cellular transformation. Oncogene 24(52):7746–7755 16299534 10.1038/sj.onc.1209038

[30] Ryan BM, Robles AI, Harris CC (2010) Genetic variation in microRNA networks: the implications for cancer research. Nat Rev Cancer 10(6):389–402 20495573 10.1038/nrc2867 PMC2950312

[31] Jegga AG, Schneider L, Ouyang X, Zhang J (2011) Systems biology of the autophagy-lysosomal pathway. Autophagy 7(5):477–489 21293178 10.4161/auto.7.5.14811 PMC3127210

[32] Zhai H, Fesler A, Ju J (2013) MicroRNA: a third dimension in autophagy. Cell Cycle (Georgetown, Tex) 12(2):246–250 23255136 10.4161/cc.23273 PMC3575453

[33] Kalinowski FC, Giles KM, Candy PA, Ali A, Ganda C, Epis MR, Webster RJ, Leedman PJ (2012) Regulation of epidermal growth factor receptor signaling and erlotinib sensitivity in head and neck cancer cells by miR-7. PLoS ONE 7(10):e47067 23115635 10.1371/journal.pone.0047067 PMC3480380

[34] Webster RJ, Giles KM, Price KJ, Zhang PM, Mattick JS, Leedman PJ (2009) Regulation of epidermal growth factor receptor signaling in human cancer cells by microRNA-7. J Biol Chem 284(9):5731–5741 19073608 10.1074/jbc.M804280200

[35] Kefas B, Godlewski J, Comeau L, Li Y, Abounader R, Hawkinson M, Lee J, Fine H, Chiocca EA, Lawler S et al (2008) microRNA-7 inhibits the epidermal growth factor receptor and the Akt pathway and is down-regulated in glioblastoma. Can Res 68(10):3566–3572 10.1158/0008-5472.CAN-07-6639 18483236

[36] Chou YT, Lin HH, Lien YC, Wang YH, Hong CF, Kao YR, Lin SC, Chang YC, Lin SY, Chen SJ et al (2010) EGFR promotes lung tumorigenesis by activating miR-7 through a Ras/ERK/Myc pathway that targets the Ets2 transcriptional repressor ERF. Can Res 70(21):8822–8831 10.1158/0008-5472.CAN-10-0638 20978205

[37] Yu Z, Ni L, Chen D, Zhang Q, Su Z, Wang Y, Yu W, Wu X, Ye J, Yang S et al (2013) Identification of miR-7 as an oncogene in renal cell carcinoma. J Mol Histol 44(6):669–677 23793934 10.1007/s10735-013-9516-5

[38] Capizzi M, Strappazzon F, Cianfanelli V, Papaleo E, Cecconi F (2017) MIR7-3HG, a MYC-dependent modulator of cell proliferation, inhibits autophagy by a regulatory loop involving AMBRA1. Autophagy 13(3):554–566 28059583 10.1080/15548627.2016.1269989 PMC5361610

[39] Pagliarini V, Wirawan E, Romagnoli A, Ciccosanti F, Lisi G, Lippens S, Cecconi F, Fimia GM, Vandenabeele P, Corazzari M et al (2012) Proteolysis of Ambra1 during apoptosis has a role in the inhibition of the autophagic pro-survival response. Cell Death Differ 19(9):1495–1504 22441670 10.1038/cdd.2012.27 PMC3422474

[40] Ferreira PMP, Sousa RWR, Ferreira JRO, Militao GCG, Bezerra DP (2021) Chloroquine and hydroxychloroquine in antitumor therapies based on autophagy-related mechanisms. Pharmacol Res 168:105582 33775862 10.1016/j.phrs.2021.105582

[41] Yu L, Alva A, Su H, Dutt P, Freundt E, Welsh S, Baehrecke EH, Lenardo MJ (2004) Regulation of an ATG7-beclin 1 program of autophagic cell death by caspase-8. Science 304(5676):1500–1502 15131264 10.1126/science.1096645

