# Supplementary material for: MYC-dependent MiR-7-5p regulated apoptosis and autophagy in diffuse large B cell lymphoma by targeting AMBRA1
Source: Mol Cell Biochem. 2024 Feb 23;480(1):191–202. doi: 10.1007/s11010-024-04946-w (PMC11695457; doi:10.1007/s11010-024-04946-w)
Supplement: Supplementary file 1 — Supplementary file1 (DOCX 18 KB) [file 11010_2024_4946_MOESM1_ESM.docx]

Supplementary Table 1. The oligonucleotide primers used to detect miR-7-5p and AMBRA1.

| **Primer** | **Sequence** |
| --- | --- |
| miR-7-5p | 5′- GCGTGGAAGACTAGTGATTT -3′ |
| AMBRA1 | 5′-TATCAACTCTGCCCGTTGGC-3′  5′-CTGTCCTCCATGTGGCTCTG-3′ |
| GAPDH | 5′-AGGTCGGTGTGAACGGATTTG-3′  5′-TGTAGACCATGTAGTTGAGGTCA-3′ |
| U6 | 5′-CTCGCTTCGGCAGCACA-3′  5′-AACGCTTCACGAATTTGCGT-3′ |
| universal reverse primer | 5′-GTGCAGGGTCCGAGGT-3′ |
