# Supplementary material for: MYC-dependent MiR-7-5p regulated apoptosis and autophagy in diffuse large B cell lymphoma by targeting AMBRA1
Source: Mol Cell Biochem. 2024 Feb 23;480(1):191–202. doi: 10.1007/s11010-024-04946-w (PMC11695457; doi:10.1007/s11010-024-04946-w)
Supplement: Supplementary file 2 — Supplementary file2 (DOCX 15 KB) [file 11010_2024_4946_MOESM2_ESM.docx]

Supplementary Table 2. The transfection of miRNA mimic and vector.

| **GENE** | **Sequence** |
| --- | --- |
| MiR-7-5p mimic | 5′-TGGAAGACTAGTGATTTTGTTGTT-3′  5′-CAACAAAATCACTAGTCTTCCATT-3′ |
| NC mimic | 5′-UUCUCCGAACGUGUCACGUTT-3′  5′-ACGUGACACGUUCGGAGAATT-3′ |
| MiR-7-5p inhibitors | 5′-AACAACAAAAUCACUAGUCUUCCA-3′ |
| NC inhibitors | 5′-CAGUACUUUUGUGUAGUACAA-3′ |
| siAMBRA1-1 | 5′-AGAACTGCAAGATCTACAA-3′ |
| siAMBRA1-2 | 5′-GGCCCTATGGTACTAACAA-3′ |
| siAMBRA1 NC | 5′-UUCUCCGAACGUGUCACGUTT-3′ |
| c-Myc siRNA | 5′-AACGUUAGCUUCACCAACAUU-3′ |
| c-Myc siRNA NC | 5′-AAUUCUCCGAACGUGUCACGU-3′ |
| pcDNA3.1-c-myc | atgcccctcaacgttagcttcaccaacaggaactatgacctcgactacgactcgg  tgcagccgtatttctactgcgacgaggaggagaacttctaccagcagcagcagc  agagcgagctgcagcccccggcgc |
