# Supplementary material for: MYC-dependent MiR-7-5p regulated apoptosis and autophagy in diffuse large B cell lymphoma by targeting AMBRA1
Source: Mol Cell Biochem. 2024 Feb 23;480(1):191–202. doi: 10.1007/s11010-024-04946-w (PMC11695457; doi:10.1007/s11010-024-04946-w)
Supplement: Supplementary file 3 — Supplementary file3 (DOCX 16 KB) [file 11010_2024_4946_MOESM3_ESM.docx]

Supplementary Table 3. The catalog numbers and dilutions of the antibodies.

| **Antibody** | **Vendor** | **Catalog number** | **Dilution** |
| --- | --- | --- | --- |
| AMBRA1 | Abcam | ab69501 | 1:1000 |
| LC3B | Sigma | L-7543 | 1:1000 |
| c-MYC | Abcam | ab32072 | 1:1000 |
| p-c-MYC | CST | 13748 | 1:1000 |
| mTOR | CST | 2972 | 1:1000 |
| PP2A-C | CST | 2038 | 1:1000 |
| PARP | CST | 9532 | 1:1000 |
| p62 | Proteintech | 18420-1-AP | 1:5000 |
| GAPDH | Proteintech | 60004-1-lg | 1:10000 |
| Anti-Mouse IgG | Jackson | 115-035-003 | 1:5000 |
| Anti-Rabbit IgG | Jackson | 111-035-003 | 1:2000 |
