# Supplementary figures and images for: MYC-dependent MiR-7-5p regulated apoptosis and autophagy in diffuse large B cell lymphoma by targeting AMBRA1
Source: Mol Cell Biochem. 2024 Feb 23;480(1):191–202. doi: 10.1007/s11010-024-04946-w (PMC11695457; doi:10.1007/s11010-024-04946-w)

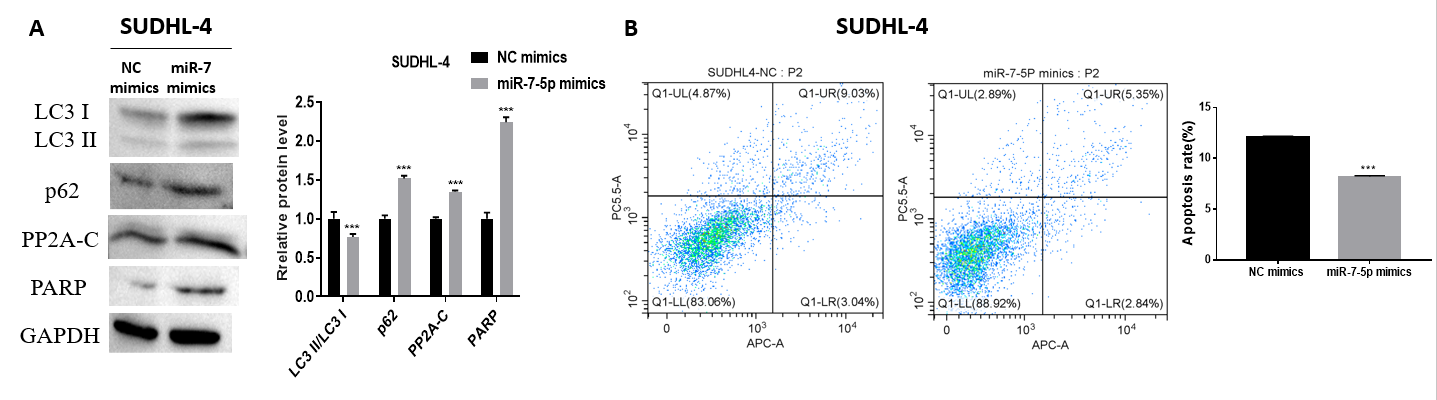

Supplement: Supplementary file 4 — Figure S1. MiR-7-5p suppresses SUDHL-4 cells autophagy and apoptosis. (A) The protein levels of autophagy and apoptosis proteins transfected with miR-7-5p mimics by western blot assays. (B) Cell apoptosis was examined using flow cytometry. Data were shown as mean ± SD. ***p < 0.001 compared with the NC group. NS indicated no significance. Supplementary file4 (TIF 252 KB) [file 11010_2024_4946_MOESM4_ESM.tif]
